# Two nearly complete genomes reveal the molecular mechanism underlying the significant fruit quality differences caused by bud sport between yellow and white‐fleshed loquat (*Eriobotrya japonica* L.)

**DOI:** 10.1111/tpj.71212

**Published:** 2026-11-04

**Authors:** Ke Zhao, Jinquan Chao, Yangang Pei, Tao Lang, Guoliang Jiang, Shuxia Sun, Hongjiang Xie, Jing Li, Meiyan Tu, Dong Chen, Mingchun Liu, Ronggao Gong, Haiyan Song

**Affiliations:** ^1^ Institute of Horticulture Research Sichuan Academy of Agricultural Sciences Chengdu 610066 China; ^2^ Key Laboratory of Horticultural Crop Biology and Germplasm Enhancement in Southwest, Ministry of Agriculture and Rural Affairs Chengdu 610066 China; ^3^ Key Laboratory of Bio‐Resource and Eco‐Environment of Ministry of Education, College of Life Sciences Sichuan University Chengdu 610065 China; ^4^ National Key Laboratory for Tropical Crop Breeding, Rubber Research Institute Chinese Academy of Tropical Agricultural Sciences Haikou 571101 China; ^5^ College of Horticulture Sichuan Agricultural University Chengdu 611134 China; ^6^ Institute of Biotechnology and Nuclear Technology Sichuan Academy of Agricultural Sciences Chengdu 610066 China

**Keywords:** loquat, bud sport, genome, molecular marker, molecular mechanism

## Abstract

Bud sport is a common and stable somatic variation in perennial fruit trees. Loquat (*Eriobotrya japonica* L.) is an evergreen fruit tree that can be categorized into yellow‐fleshed and white‐fleshed types based on the flesh color. To uncover the genetic basis, we herein assembled two nearly complete genomes for a yellow‐fleshed ‘Dongting’ loquat (TBY) and its white‐fleshed bud sport (TBW). The final sizes of assemblies were 773.29 Mb and 728.86 Mb with contig N50 of 31.87 Mb and 28.63 Mb for TBY and TBW, respectively. Comparative genomics analysis indicated that the heterozygosity ratio of TBY was nearly two‐fold higher than that of TBW (0.53% vs. 0.27%). Integrated multi‐omics and molecular marker detection results revealed that homozygosity of the *EjPSY2A* ^*d*^/*EjPSY2A* ^*d*^ allele is the cause of the change in flesh color from yellow to white in TBY. Additionally, *EjSUSs* (*−1*, *−2*, *−3*, *−12*) and *EjSPS3* might be the key genes responsible for the significant accumulation of hexoses and sucrose, respectively, in the flesh of TBY and TBW. Furthermore, high expression of *EjC4H8* and *Ej4CLs* (*−7*, *−10*, *−20*) in TBY might lead to the accumulation of more lignin in its flesh. Taken together, these findings provide valuable insights into the molecular mechanism differences between yellow‐ and white‐fleshed loquat and highlight the significance of bud sport as a valuable approach for generating novel germplasm resources in fruit trees.

## Significance Statement

In the present study, we integrate the analysis of the genome, transcriptome, and metabolome to reveal the insightful genetic information underlying the significant fruit quality differences caused by bud sport between yellow‐fleshed Dongting loquat (TBY) and its white‐fleshed bud sport (TBW). These findings provide valuable insights into the molecular mechanism difference between yellow‐ and white‐fleshed loquat and highlight the significance of bud sport as a valuable approach for generating novel germplasm resources in fruit trees.

## INTRODUCTION

Somatic mutation is a common phenomenon leading to the genetic variation, which is shared by both annual crops and perennial plants. Generally, the mutation rate of ‘active cell’ is higher than that of ‘resting cell’ (Moore et al., 2021). Meristem are a small pool of active cells with pluripotency. There are mainly three kinds of meristems in plants: the shoot meristems, the root meristems, and the vascular meristems (Eshed, 2021; Lindsay et al., 2024). These meristems endow plants with the ability to continuously produce new organs such as leaf, axillary branch, flower primordia, root, phloem, xylem, etc (Wang et al., 2018). The mutation of meristems usually leads to the emergence of novel traits different from the maternal plant, termed as ‘bud sport’ (Jiang, Wang, et al., 2020; Liu et al., 2022). Because of bud sport provide new phenotype while retaining the elite qualities of the maternal plant, it is very important for the breeding of new cultivar, especially in the asexual reproduction of trees and horticultural crops. For instance, 1337 and 863 bud sport mutants are recorded in sweet orange and apple in the past 80 years (Wang, Huang, et al., 2021; Zhang et al., 2019).

Bud sport often occurs in unfavorable external environments such as ultraviolet radiation, high/low temperature, water availability, etc. (Ban & Jung, 2023). Over the past decades, researchers have conducted a large number of studies on the molecular mechanism of bud sport and achieved remarkable results. Research findings from a study utilizing ‘Fuji’ apples and their 74 somatic variants demonstrate that spur‐type and early‐maturation traits observed in ‘Fuji’ sport varieties originate from multiple independent events (Cai et al., 2024). Recent studies leveraging comparative genomics and multi‐omics analyses have indicated that loss of heterozygosity (LOH), allelic dropout (ADO), and allele‐specific expression (ASE) may constitute important drivers of fruit quality variations in bud‐sport materials of apple (Sun et al., 2024) and peach (Zhou et al., 2023). However, the inherent molecular mechanism underlying the significant fruit quality differences caused by bud sport is still poorly understood at the whole‐genome level.

Loquat (*Eriobotrya japonica* L.) is an evergreen fruit tree belonging to the Rosaceae family. Based not only on fruit color but also on sweetness and firmness, loquat cultivars are categorized into yellow‐fleshed and white‐fleshed types (Lin, 2019). The yellow‐fleshed loquats present orange or orange‐red colors derived primarily from their abundant carotenoids (Jiang et al., 2015; Zou et al., 2020). The white‐fleshed loquats attract consumers with their delicate creamy white flesh, tender consistency, and succulent sweetness, which grants them a high degree of consumer favoritism based on their superior flavor profile and delectable taste (Chen et al., 2025). It is generally believed that the white‐fleshed loquat mutates from the yellow‐fleshed loquat, but the inherent genetic mechanism has not been well understood (Li et al., 2017; Zou et al., 2020). In the past decade, researchers are attempting to uncover the molecular mechanisms underlying the differences between yellow and white‐fleshed loquat cultivars from multiple perspectives. Fu et al. (2014) reported that *EjPSY2A* ^*d*^ (phytoene synthase) lacked the C‐terminal region and corresponding catalytic activity, which might account for the absence of carotenoids in white‐fleshed loquat. Wang and Paterson (2021) identified hundreds of flesh color‐related genes by performing genome‐wide association study analysis.

Previously, we reported that a bud sport derived from the yellow‐fleshed ‘Dongting’ loquat (termed as TBY) exhibited an appealing white flesh phenotype (termed as TBW) (Sun et al., 2013). TBW differs significantly from TBY in terms of sugar component, flesh texture, and cold resistance, thus underlining its immense potential for breeding purposes (Chen et al., 2025; Pan et al., 2020; Song et al., 2024). Further high‐density molecular marker detection and continuous years of follow‐up observation on grafted progenies confirmed the stable nature of the bud sport of TBW (Sun et al., 2012; Sun et al., 2017).

Bud sport is a critical source for generating novel traits in fruit trees, and dissecting the molecular mechanism underlying bud sport‐induced trait variations will facilitate the better utilization of this breeding approach. In the present study, we integrate the analysis of the genome, transcriptome, and metabolome to reveal the insightful genetic information underlying the significant fruit quality differences caused by bud sport between TBY and TBW (Figure S1). Our findings provide valuable insights into the molecular mechanism difference between yellow‐ and white‐fleshed loquat and highlight the significance of bud sport as a valuable approach for generating novel germplasm resources in fruit trees.

## RESULTS

### Genome assembly, annotation, and comparative analysis

The bud sport (TBW) and its maternal plant (TBY) were identified in Aba Tibetan and Qiang Autonomous Prefecture, Sichuan Province (Figure 1a,b). Based on survey results, the genome size of TBY and TBW was estimated to be 750.60 Mb and 724.27 Mb (Figure S2; Table S1), which was comparable to 749.15 Mb for TBY and 732.87 Mb for TBW obtained by flow cytometry (Figure S3; Table S2). Interestingly, the heterozygosity ratio of TBY was 0.53%, which was nearly double that of TBW (0.27%). Though the integration of 56.97 Gb of PacBio CCS (circular consensus sequencing) sequences and 167.72 Gb high‐throughput chromatin conformation capture (Hi‐C) data (Tables S3–S5), the final genome size of TBY and TBW was 773.29 Mb (745.24 Mb anchored onto 17 pseudomolecules) and 728.86 Mb (719.39 Mb anchored onto 17 pseudomolecules), with contig N50 values of 31.87 Mb and 28.63 Mb, respectively (Figure 1c,d; Table S6). Only 33 gaps were present in the TBY assembly and 30 gaps in the TBW assembly, averaging nearly two gaps per chromosome (Figure S4). Benchmarking Universal Single‐Copy Orthologs (BUSCO) analysis revealed 99.13% and 99.19% of the core eukaryotic genes were found to be complete in the TBY and TBW assemblies (Table S7). These results indicate that the two nearly complete genomes represent a significant improvement in the loquat genome compared to the previous versions (Table S8).

**Figure 1:**
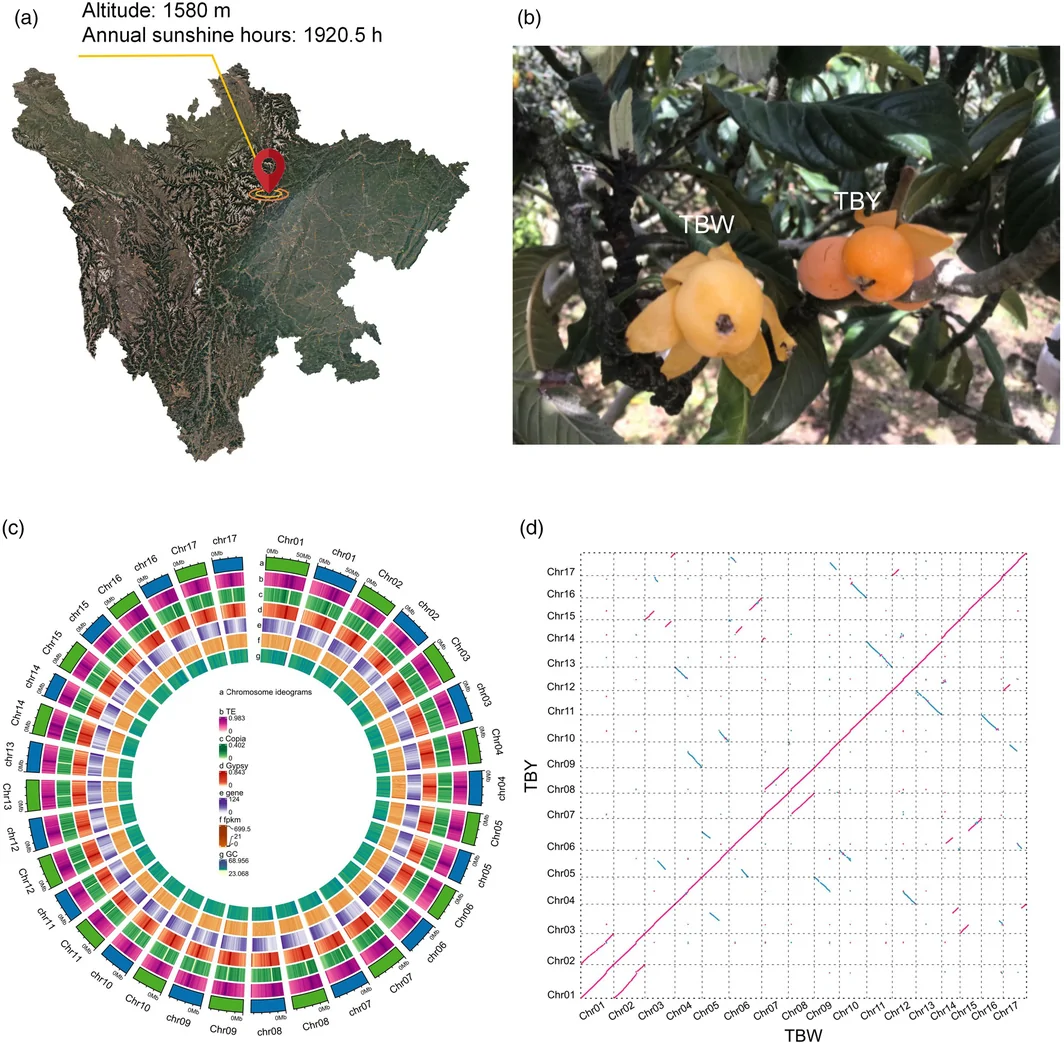
The origin of TBY and TBW, as well as the comparison of the characteristics between the two genomes. (a) The altitude and annual sunshine duration of the orchard where TBW was identified. (b) TBW is a major variant branch derived from TBY. (c) Genomic characteristics analysis of TBY (Chr) and TBW (chr). 1, pseudochromosome. 2, TE density. 3, TE density of copia type. 4, TE density of gypsy type. 5, gene density. 6, the highest FPKM value in all tissues. 7, GC content. (d) The illustration of dot plots displays synteny regions between the TBY genome and the TBW genome. The horizontal axis represents the reference coordinate, and the vertical axis represents the query coordinate. Red dots signify collinear regions, while blue dots indicate reverse alignments, which have been revised from the TBY genome.

Genome annotation indicated 58.41% (451.64 Mb) of the TBY assembly and 54.97% (400.62 Mb) of the TBW assembly were repeat sequences (Table S6). By combining homology prediction, *de novo* prediction and transcriptome prediction, a total of 47 530 and 43 915 protein‐coding gene models were respectively annotated in the TBY and TBW assembles (Table S9). Approximately 99.05% and 99.29% of the identified genes were functionally annotated via similarity searches of homologous sequences and protein domains in both assemblies (Table S10). Furthermore, our analysis further identified a multitude of non‐coding RNA genes, including 16 576 rRNA, 3707 tRNA, 122 miRNA and 114 snRNA genes in TBY, as well as 14 277 rRNA, 1905 tRNA, 197 miRNA and 252 snRNA genes in TBW.

Using the TBY assembly as a reference, a total of 30 inversions, 271 translocations, 101 reference‐specific duplications, 897 290 single nucleotide polymorphisms (SNPs) and 135 466 insertions and deletions (Indels) were identified in the TBW assembly (Figure S5, Tables S11 and S12). To investigate whether genomic regions exhibiting loss of heterozygosity (LOH) are present in the TBW genome, we performed a genome‐wide LOH analysis based on heterozygous SNP distribution. A total of 1 247 582 heterozygous SNPs and 1461 LOH blocks were identified in TBW, covering approximately 335.4 Mb (Table S13). We then independently verified 16 out of the 20 randomly selected SNPs (validation rate of 80%) by Sanger sequencing (Figure S6, Table S14). Kyoto Encyclopedia of Genes and Genomes (KEGG) analysis of SNPs or Indels related genes showed several similar pathways were enriched, including “fatty acid elongation”, “diterpenoid biosynthesis”, “isoflavonoid biosynthesis”, “benzoxazinoid biosynthesis”, “sesquiterpenoid and triterpenoid biosynthesis”, and “cyanoamino acid metabolism”, etc (Figure S7).

### Comparative transcriptome analysis of key periods between TBY and TBW

TBY and TBW share identical phenological stages. Before and after flesh discoloration are the key periods for loquat fruit quality (Song et al., 2024). To gain insight into the molecular mechanism between TBY and TBW, three time‐point samples were collected at 143 days after pollination (DAP), 150 DAP, and 155 DAP (termed as S1, S2, and S3 stage), and performed by transcriptomic analysis (Figure 2a; Table S15). Under the threshold of log_2_|Fold Change| ≥1 and false discovery rate (FDR) <0.01, a total of 1678 (735 up and 943 down), 3055 (1254 up and 1801 down), and 4299 (2170 up and 2129 down) differentially expressed genes (DEGs) were respectively detected at S1, S2, and S3 stages (Table S16). This result indicated the difference gradually increases at the transcription level with the fruit ripening, as according to the trends displayed by the principal component analysis (PCA) (Figure 2b).

**Figure 2:**
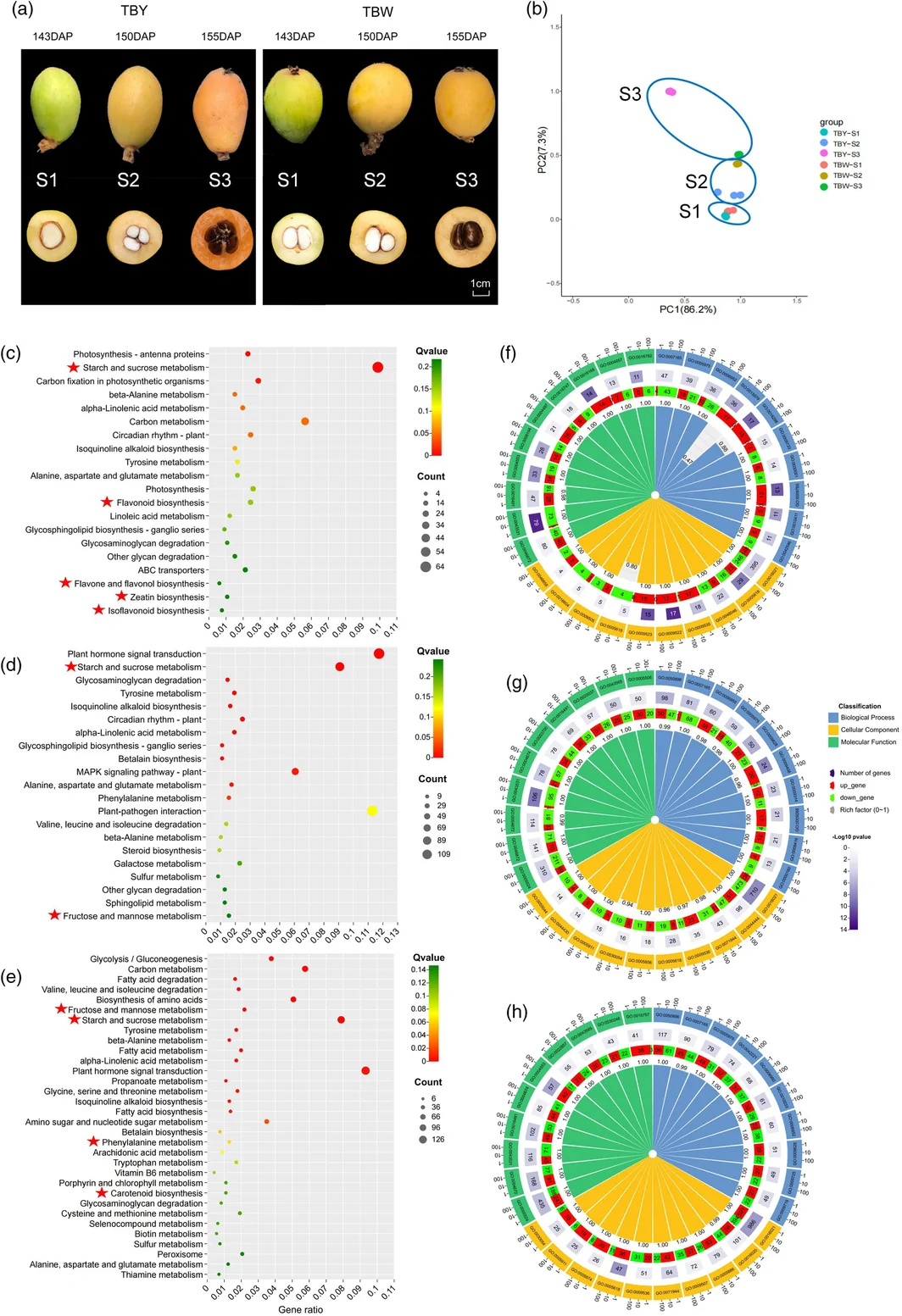
Analysis of transcriptome data of TBY and TBW at different developmental stages of fruits. (a) The phenotypes of the flesh and peel of TBY and TBW were recorded at 143 days, 150 days and 155 days after pollination, termed as S1, S2, and S3, respectively. (b) Results of principal component analysis of the transcriptome data. (c–e) KEGG pathway enrichment analysis of the DEGs between TBY and TBW from the S1 to S3 stages. (f–h) GO enrichment analysis of top 30 terms of the DEGs between TBY and TBW from the S1 to S3 stages. The KEGG pathways of primary interest in this study are marked with red five‐pointed stars.

To better understand the inherent information, all DEGs were grouped by both KEGG and Gene Ontology (GO) analysis (Figure 2c–h). KEGG analysis identified numerous pathways significantly enriched in different flesh discoloration stages. At the S1 stage, the enriched pathways included “starch and sucrose metabolism”, “flavonoid biosynthesis”, “flavone and flavonol biosynthesis”, “zeatin biosynthesis”, “isoflavonoid biosynthesis” (Figure 2c). At the S2 stage, the enriched pathways included “starch and sucrose metabolism”, “phenylalanine metabolism”, “gluctose and mannose metabolism” (Figure 2d). At the S3 stage, the enriched pathways included “fructose and mannose metabolism”, “starch and sucrose metabolism”, “phenylalanine metabolism”, “carotenoid metabolism” (Figure 2e).

Biological process is the most important term among the three GO categories. We detected the enriched terms such as “carbohydrate metabolic process (GO:0005975)”, “defense response (GO:0006952)”, “cell wall biogenesis (GO:0042546)” at the S1 stage (Figure 2f; Data S1); “response to stress (GO:0006950)”, “carbohydrate metabolic process (GO:0005975)”, “response to abiotic stimulus (GO:0009628)”, “response to heat (GO:0009408)”, “response to radiation (GO:0009314)”, “response to temperature stimulus (GO:0009266)” at the S2 stage (Figure 2g, Data S2); “carbohydrate metabolic process (GO:0005975)”, “response to stimulus (GO:0050896)”, “response to abiotic stimulus (GO:0009628)” at the S3 stage (Figure 2h; Data S3).

### The core carotenoid components that lead to the color change of loquat flesh

Previous study reported that carotenoids were the main pigments causing the difference of flesh color between yellow and white‐fleshed loquat cultivars (Liu et al., 2016). The hue angle, also known as H°, has been widely recognized as an indicator of maturity in numerous plant fruits and decreases with increasing carotenoids (Medveckienė et al., 2023; Song, Liu, et al., 2022). In the present research, detection results showed that the total carotenoids increased gradually with fruit development, and β‐carotene was significantly negatively correlated with the hue angle (Figure 3a; Figure S8). A total of 12 kinds of carotenoid components were identified by employing ultra‐performance liquid chromatography‐mass spectrometry (UPLC‐MS), including β‐cryptoxanthin and its derivatives, β‐carotene and its derivatives, rubixanthin and its derivatives, violaxanthin and its derivatives, and E/Z‐phytoene (Figure 3b). Among them, the differences in the E/Z‐phytoene and β‐carotene contents between TBY and TBW were the largest, both exceeding more than 10‐fold (Data S4). Abscisic acid (ABA) is one of the degradation products of carotene and NCED is the rate‐limiting enzyme for ABA biosynthesis. In the present study, ABA content was subsequently higher in TBW than that in TBY (Figure 3c).

**Figure 3:**
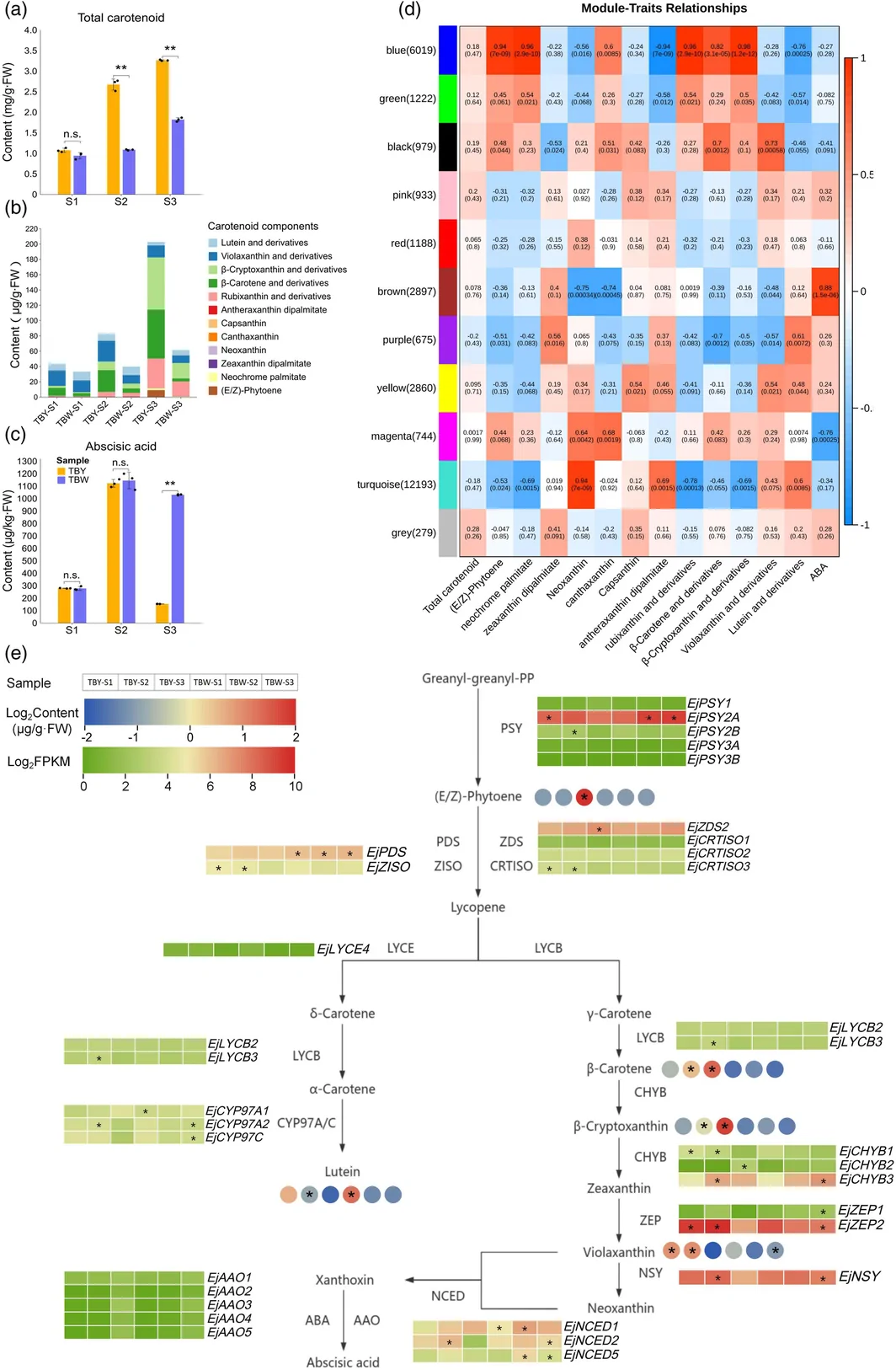
A combined analysis of the transcriptome and carotenoid‐targeted metabolome of TBY and TBW during fruit development. (a) The total carotenoid content of the flesh of TBY and TBW were measured during fruit development. One or two asterisks indicate statistical significance by Student's *t*‐test at 0.05 or 0.01 levels, respectively. N.S. indicates not significant, and this notation applies hereinafter. (b) Stacked‐bar plot of 12 main carotenoid components during fruit development. (c) The content of abscisic acid in the flesh of TBY and TBW during fruit development. (d) Hierarchical clustering dendrogram of modules based on transcriptome data and major carotenoid compounds. (e) The key differential metabolites and expressed genes mapped to the carotenoid metabolic pathway in TBY and TBW were identified. Circles represent metabolites, and squares represent genes. One asterisk indicates statistical significance at the 0.05 level according to Student's *t*‐test.

To detect the putative key genes regulating carotenoid metabolism, we conducted a weighted gene co‐expression network analysis (WGCNA) with transcriptome data and key substances in the carotenoid metabolic pathway (Figure 3d). PSY is the first enzyme of carotene biosynthesis, catalyzing geranylgeranyl‐PP to form phytoene. We detected five *PSY* isoforms in the transcriptome, and *EjPSY2A* was the specific one highly expressed in the flesh (Data S5). The expression of *EjPSY2A* was notably higher in TBW than that in TBY at the S2 and S3 stages, but the content of E/Z‐phytoene was significantly higher in TBY at the S3 stage (Figure 3e).

Sequence analysis showed a 321 bp deletion of *EjPSY2A* caused a premature termination code in TBW, which was identical to *EjPSY2A* ^*d*^ reported previously (Fu et al., 2014) (Figure 4a,b; Figure S9). We utilized the molecular markers for flesh color in loquat that were developed in our earlier research (Song, Sun, et al., 2022). The test results indicated that the *EjPSY2A* gene exhibited a heterozygous state in TBY but a homozygous state in TBW (Figure 4c). Quantitative real‐time polymerase chain reaction (RT‐qPCR) analysis showed a high degree of consistency (Spearman's *ρ* = 0.79) with the RNA‐Seq data, which verified the accuracy of the RNA‐Seq results (Figures S10 and S11). Subsequently, the *EjPSY2A* gene was transiently overexpressed in the flesh of TBW, and the results demonstrated that *EjPSY2A* could restore the accumulation of β‐carotene in the flesh of TBW (Figure 4d–f). Meanwhile, transient overexpression of the *EjNCED2* gene in the flesh of TBY instead reduced the accumulation of β‐carotene in the flesh of TBY (Figure 4g–i).

**Figure 4:**
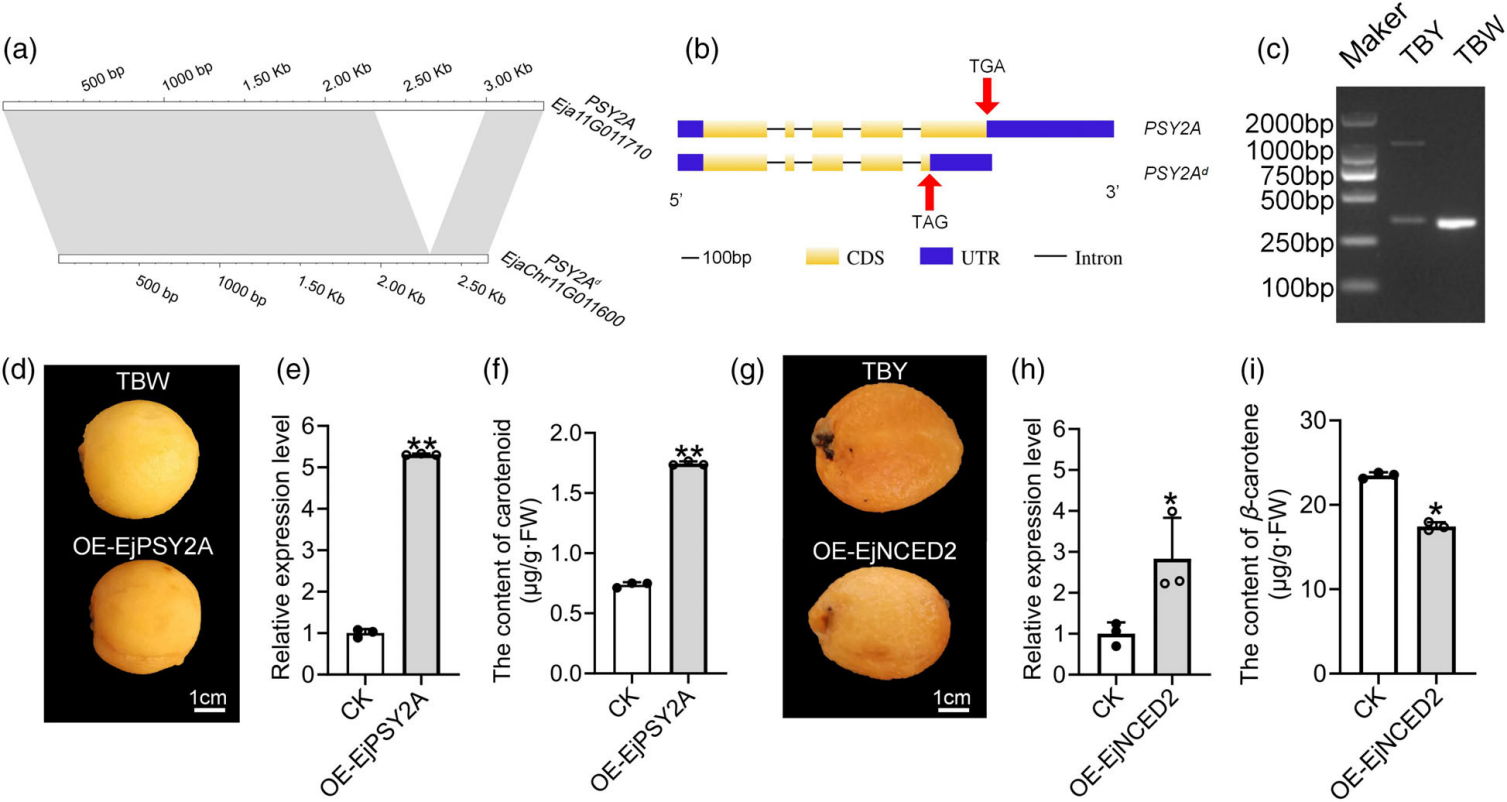
Comparative genomics identifies key genes governing carotenoid accumulation in loquat, followed by molecular marker screening and functional verification. (a, b) The gene structure revealed that there was a 321 bp deletion in the 5th exon region of TBW compared to TBY, resulting in a change in the termination codon from TGA to TAG, thereby causing premature termination of translation. (c) *EjPSY2A* was genotyped using previously published molecular markers, and TBY had both large and small fragments, whereas in TBW, only a small fragment was found. Phenotypic changes (d), gene expression levels (e), and β‐carotene content (f) following transient overexpression of the *EjPSY2A* and *EjPSY2A* ^*d*^ genes in the flesh of TBW. Phenotypic changes (g), gene expression levels (h), and β‐carotene content (i) following transient overexpression of the *EjNCED2* in the flesh of TBW. One or two asterisks indicate statistical significance by Student's *t*‐test at 0.05 or 0.01 levels, respectively.

### The dynamic of sugar components between TBY and TBW

In the previous study, we demonstrated the sugar content was higher in TBW compared to that in TBY (Li et al., 2017). To further exemplify the key components contributing to the taste differences between TBY and TBW, we conducted sugar metabolism at the S1, S2, and S3 stages, respectively. The PCA results indicated that the significant changes in the sugar components in the flesh were at the S3 stage (Figure 5a). A total of eight components were detected: sucrose (Suc), fucose (Fuc), glucose (Glu), fructose (Fru), rhamnose (Rha), xylitol, inositol, and sorbitol (Figure 5b; Data S6). Among them, Suc, Fru, Glu, and sorbitol constituted the four major sugar components in loquat flesh. With the ripening of the fruit, a decrease in the sorbitol content and an increase in both the Fru as well as the Glu contents were observed in both samples, and there was a significant difference (*P* < 0.01) in the Suc, Fru, and Glu contents at the S3 stage (Figure 5c).

**Figure 5:**
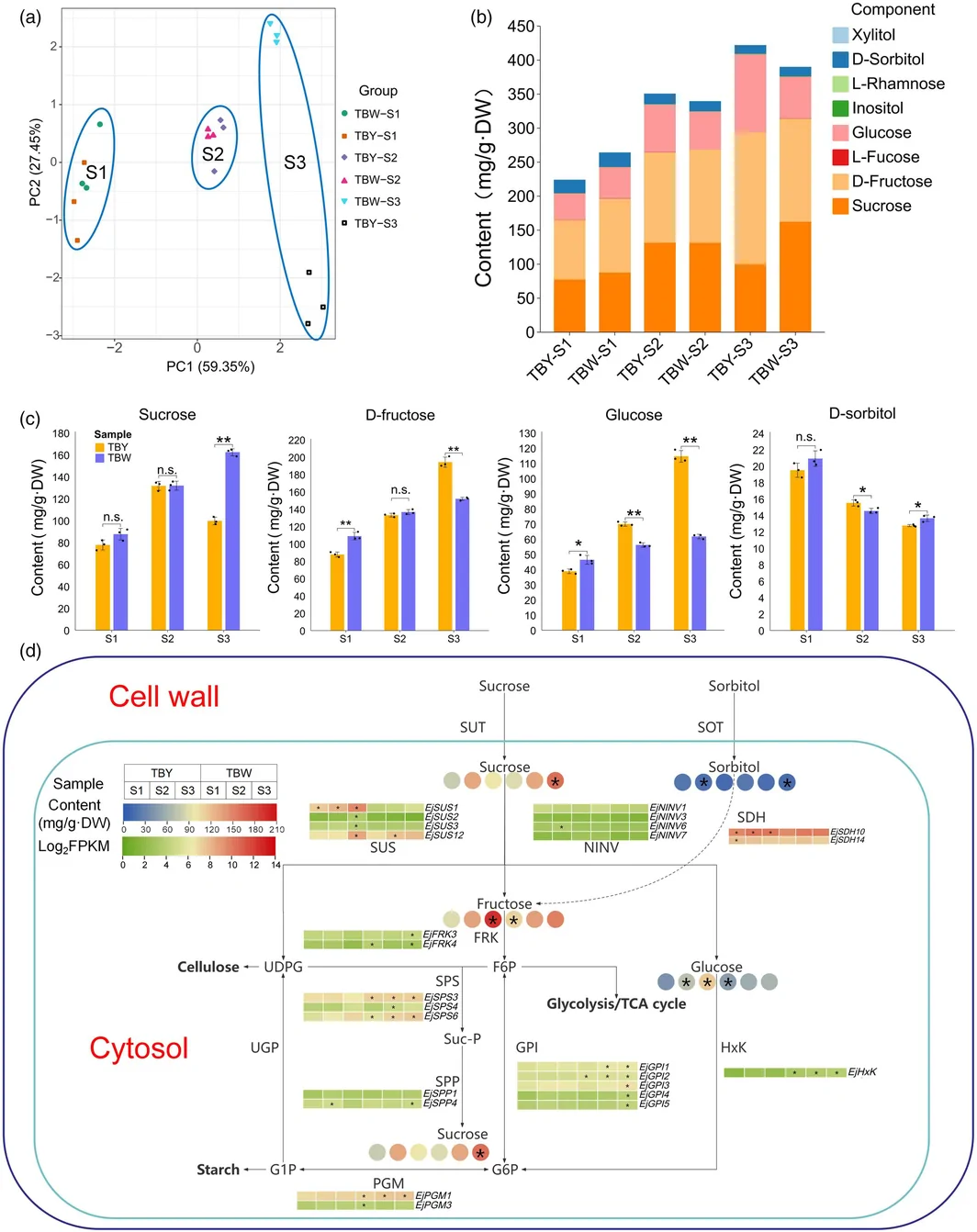
A combined analysis of the transcriptome and sucrose‐targeted metabolome of TBY and TBW during fruit development. (a) Results of the principal component analysis of the sucrose‐targeted metabolome data. (b) Stacked‐bar plot of the sugar components. (c) Histogram of the four main sugar components in the loquat flesh. One or two asterisks represent statistical significance by Student's *t*‐test at 0.05 or 0.01 levels, respectively. N.S. indicates not significant, and this notation applies hereinafter. (d) Integrated analysis of the key differential metabolites and expressed genes associated with the sugar metabolic pathway of TBY and TBW. Circles represent metabolites, and squares represent genes. One asterisk indicates statistical significance at the 0.05 level according to Student's *t*‐test.

To detect the putative key genes, a combined analysis of the transcriptome and sugar metabolism was performed (Figure 5d; Data S7). Sucrose synthase (SUS) is essential for sucrose degradation, while sucrose phosphate synthase (SPS) is crucial for sucrose biosynthesis. Here, we showed that four *EjSUSs* (*−1*, *−2*, *−3*, *−12*) were highly expressed in TBY while three *EjSPSs* (*−3*, *−4*, *−6*) were highly expressed in TBW. Hexokinase is a rate‐limiting enzyme for Glu degradation. We detected that *EjHxK* was highly expressed at all three stages in TBW. Sorbitol dehydrogenase (SDH) is the key enzyme catalyzing sorbitol to Fru, while fructokinase (FRK), glucose‐6‐phosphate isomerase (GPI), and phosphoglucomutase (PGM) are the three enzymes for Fru degradation. Presently, we found two *SDH* (*EjSDH10* and *EjSDH14*) were highly expressed in TBY while two *FRK* (*EjFRK3* and *EjFRK4*), five *GPI* (*EjGPI‐1, −2, −3, −4, −5*), and two *PGM* (*EjPGM1* and *EjPGM3*) were highly expressed in TBW, respectively (Figure 5d). The correlation analysis between the RNA‐Seq data and the RT‐qPCR expression levels showed a high degree of consistency (Spearman's *ρ* = 0.79), which verified the accuracy of the RNA‐Seq results (Figures S12 and S13).

### The dynamic of phenylpropanoids components between TBY and TBW

Phenylpropanoid compounds, such as anthocyanins, flavonols, and lignin, are a significant class of secondary metabolites regarding fruit quality. Unlike sugar and carotenoid, the PCA results showed that the difference of phenylpropanoid between the two materials was gradually decreased along with the development of fruit (Figure 6a). A total of 12 phenylpropanoid compounds were identified in loquat flesh by using targeted metabolomics technology (Figure 6b; Data S8). Among them, proanthecyanidin and epicatechin were the two most abundant metabolites, accounting for over 90%. Interestingly, syringaldehyde, a product of lignin degradation, was specifically detected in TBY. Through the combined analysis of targeted metabolomics and transcriptome, we detected *EjC4Hs* (*EjC4H7* and *EjC4H8*) and *Ej4CLs* (*−7*, *−10*, *−20*), the key genes involved in lignin biosynthesis, that were highly expressed in TBY (Figure 6c; Data S9). The results of RT‐qPCR showed a high degree of consistency (Spearman's *ρ* = 0.76) with the transcriptome results, and *EjC4H8* in TBY was significantly higher than those in TBW at S3 stage (Figures S14 and S15). Subsequently, histochemical staining and lignin determination confirmed the higher lignin content in TBY flesh compared to that in TBW (Figure 6d,e), which might be accounted for by TBY fruit having more firmness than TBW (Figure 6f).

**Figure 6:**
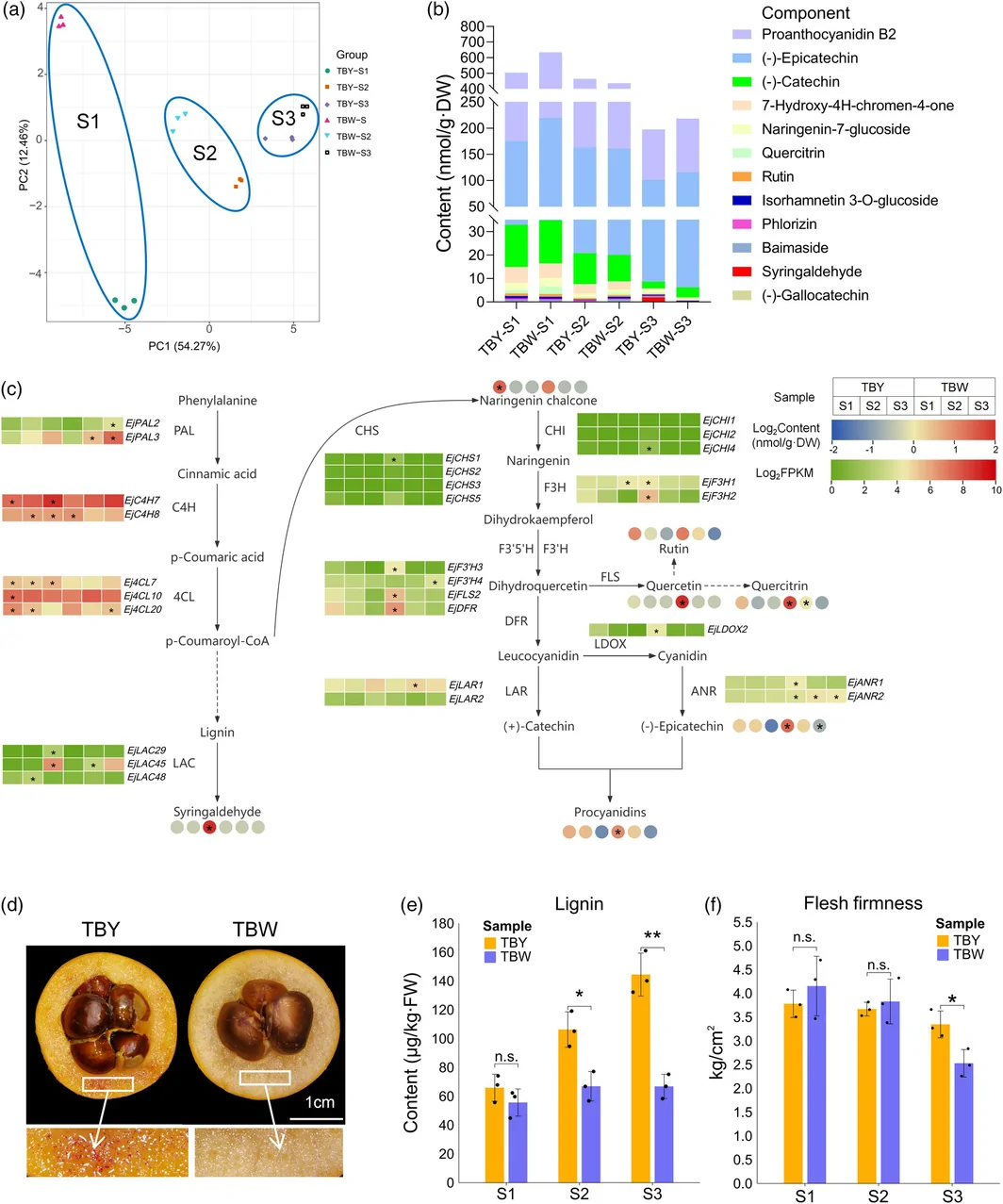
A combined analysis of transcriptome and phenylpropanoid‐targeted metabolome of TBY and TBW during fruit development. (a) Results of the principal component analysis of the phenylpropanoid‐targeted metabolome data. (b) Stacked‐bar plot of the top 12 phenylpropanoid components during fruit development. (c) Integrated analysis of key differential metabolites and expressed genes involved in the phenylpropanoid metabolic pathway of TBY and TBW. Circles represent metabolites, and squares represent genes. One asterisk indicates statistical significance at the 0.05 level according to Student's *t*‐test. (d) Detection of lignin by phloroglucinol in the mature fruits of TBY and TBW. (e) Lignin content and fruit firmness of TBY and TBW at different developmental stages. One or two asterisks represent statistical significance by Student's *t*‐test at 0.05 or 0.01 levels, respectively. N.S. indicates not significant, and this notation applies hereinafter.

## DISCUSSION

A complete genome holds great potential to unveil the missing genetic information that is essential for crop breeding purposes. With the advancement of genome sequencing and assembly, more and more genomes have been upgraded to the complete versions in recent years (Zhou et al., 2022). Though four loquat genome versions have been published previously (Jiang, An, et al., 2020; Jing et al., 2023; Su et al., 2021; Wang, 2021), the quality of the genome still has potential for improvement. In the present study, we assembled two nearly complete loquat genomes with an average of two gaps per chromosome. The contig N50 values of the two cultivars range from 28.63 Mb to 31.87 Mb, increasing more than 5.64‐fold to 6.27‐fold compared to the best version of the genome published previously. The two near‐complete genomes assembled here will undoubtedly accelerate loquat molecular breeding in the future.

Although we have previously confirmed that TBW is a bud sport derived from TBY through phenotypic comparison and molecular marker approaches, the substantial differences in flesh color, flavor, and texture between the two accessions still cannot be effectively explained (Chen et al., 2025; Song et al., 2024; Sun et al., 2012; Sun et al., 2017), which prompted us to further explore the variations in their genetic information via comparative genomics. Interestingly, genome survey analysis shows that the heterozygosity ratio of TBY is 0.53%, which is nearly twice that of its bud sport TBW (0.27%). Subsequent loss of heterozygosity (LOH) analysis further confirmed the presence of abundant LOH regions in the TBW genome, which resembles the mutation mechanisms recently reported for bud‐sport materials of apple and peach (Sun et al., 2024; Zhou et al., 2023). A recent study on the genus *Carya* has broadly explained the cause of LOH events in perennial tree genomes: to adapt to environments and maintain genome stability, LOH can expose recessive deleterious genes, making them eliminated via purifying selection (Zhang et al., 2026). The present study supports the environmental adaptation strategy mediated by LOH‐driven bud sports in loquat. Further exploration of the loci under purifying selection in future research will help unravel the genetic basis of quality differences among loquat bud sport accessions.

Unfavorable external environments, such as ultraviolet radiation, high/low temperature, and water availability, are the main reasons for bud sport formation, which mainly leads to genetic variations in meristem cells including DNA replication/repair error, transposable elements activation, epigenetic variations, etc (Lou et al., 2022; Ramu et al., 2017; Zeng et al., 2020). Failures in the DNA replication process and damage caused by chemicals or radiation may produce DNA double‐strand breaks (DSBs) (Wyrick & Roberts, 2015). Homologous recombination (HR) and non‐homologous end joining (NHEJ) are two ways to repair DSBs in eukaryotic organisms (Li et al., 2023). For HR, the intact homologous chromosome acts as template. The process does not produce new genes but can lead to the loss of heterozygosity (Zhang et al., 2023). For NHEJ, the two broken ends are re‐ligated by ligase, and small insertions or deletions (indels) are often made at the lesion site (Chen et al., 2021). Notably, the Sichuan Tibetan region, renowned for its high altitude, intense ultraviolet radiation, and substantial diurnal temperature fluctuations, subjects plants to extreme conditions that uniquely shape their biological characteristics (Wang, Liu, et al., 2021; Xia et al., 2018), gene expression patterns (Li et al., 2024; Zhang et al., 2024), and even chromosome configurations (Guo et al., 2020; He et al., 2020). In fact, we also notice some stress‐related GO terms such as “response to abiotic stimulus (GO:0009628)”, “response to heat (GO:0009408)”, “response to radiation (GO:0009314)”, “response to temperature stimulus (GO:0009266)” are specifically enriched in DEGs. We therefore speculate that HR for the DSBs generated by unfavorable external environments could directly underlie the LOH‐driven bud sport event in TBW.

It is generally believed that the white‐fleshed loquat mutates from the yellow‐fleshed loquat (Li et al., 2017; Zou et al., 2020). Carotenoids are one of the important components that endow fruit flesh color (Wang et al., 2023). Fu et al. (2014) showed the content of β‐carotene is significantly higher in yellow‐fleshed loquat cultivars than that in white‐fleshed loquat cultivars, and identified a homozygous non‐functional variant of *EjPSY2A* ^*d*^/*EjPSY2A* ^*d*^ in white‐fleshed loquat cultivars originating from different regions. In 2022, a larger scale loquat cultivars experiment confirms *EjPSY2A* ^*d*^/*EjPSY2A* ^*d*^ exists in white‐fleshed loquat cultivars, while *EjPSY2A*/*EjPSY2A* or *EjPSY2A*/*EjPSY2A* ^*d*^ exists in yellow‐fleshed loquat cultivars (Song, Sun, et al., 2022). In the present study, we demonstrate that the *EjPSY2A*/*EjPSY2A* ^*d*^ locus resides within an LOH region on chromosome 11 (Figure S16), and provide direct evidence that the *EjPSY2A*/*EjPSY2A* ^*d*^ in the yellow‐fleshed loquat TBY mutates to the *EjPSY2A* ^*d*^/*EjPSY2A* ^*d*^ in the white‐fleshed loquat TBW possible through HR for the DSBs generated by unfavorable external environments in Sichuan Tibetan region. This result clarifies the origin of white‐fleshed loquat and has important application value in loquat molecular breeding.

In this study, comparative genomic and transcriptome analyses identified a substantial number of SNPs/Indels and DEGs, and we further anchored these key DEGs to the carotenoid metabolism, sugar metabolism and phenylpropanoid metabolism pathways related to loquat quality differences via targeted metabolomics. Although transient expression assays in loquat flesh have confirmed the gene function of *EjPSY2A*/*EjPSY2A* ^*d*^, the functions of more variant loci remain to be further verified. Interestingly, despite the TBW‐specific C‐terminal truncation altering the protein encoded by *EjPSY2A*, its transcript abundance is not reduced, and the elevated expression level may represent a compensatory transcriptional response to the decreased carotenoid biosynthesis. In addition, allele‐specific expression (ASE) could also account for the large number of DEGs between TBY and TBW. Recently, research suggests that the imbalance between the expression levels of alleles has been suggested as a mechanism of elite traits formation (Abdullah et al., 2024). In apple, Sun et al. (2020) report 19% of genes, including many associated with fruit quality, display allele‐specific expression at 13 developmental stages. Considering that the heterozygous of TBW is significantly reduced compared to TBY, we speculate the SNPs/Indels detected here might be the differences between homologous chromosomes, which further causes the imbalance expression of allele genes. Assembling the haplotype‐resolved genomes of TBW and TBY will be the next focus of the research.

In summary, we assembled two nearly complete genomes for a yellow‐fleshed ‘Dongting’ loquat and its white‐fleshed bud sport. We speculate that the unfavorable external environment in the high‐altitude areas of western Sichuan is the reason for bud sport: DSBs caused by the external environment lead to a decrease in genome heterozygosity during HR repair, resulting in the allele‐specific expression related to loquat color and quality (Figure 7).

**Figure 7:**
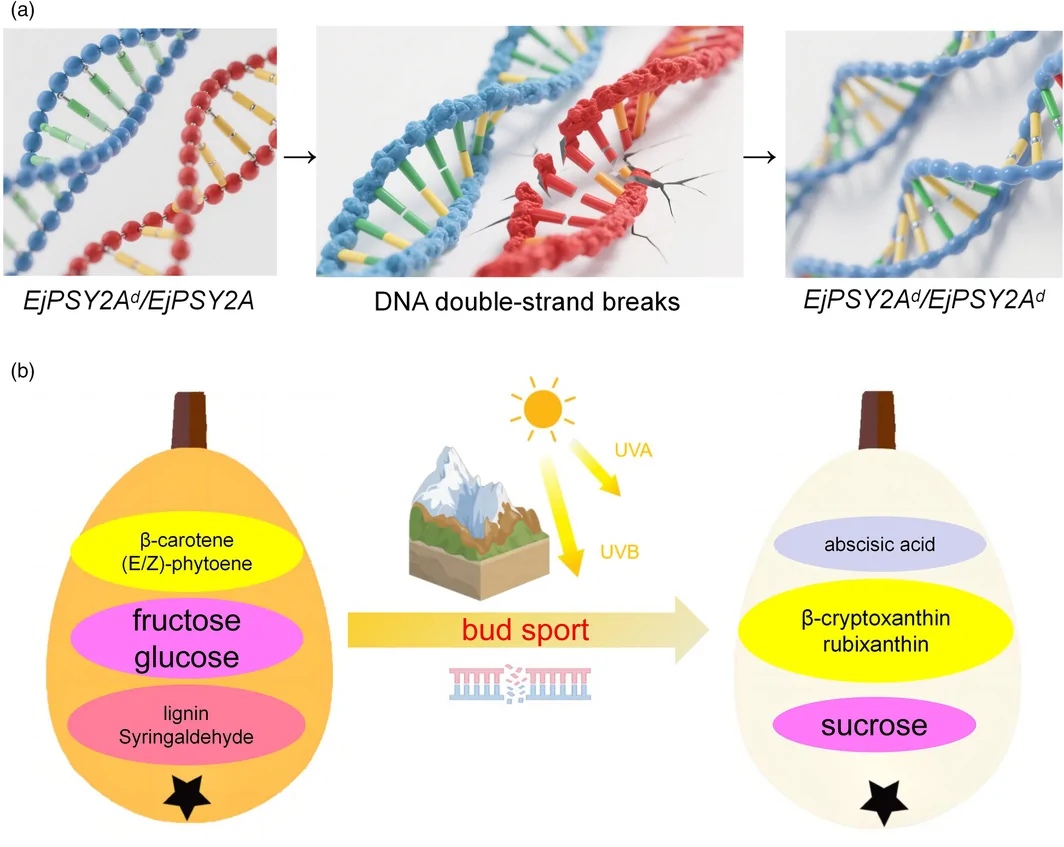
The model of bud sport leading to alterations in significant quality traits of loquat. (a) In the yellow‐fleshed loquat TBY, DNA double‐strand breaks (DSBs) in *EjPSY2A* ^*d*^ */EjPSY2A* genes triggered template‐dependent repair using *EjPSY2A* ^*d*^ as a template, resulting in homozygous *EjPSY2A* ^*d*^ */EjPSY2A* ^*d*^ mutations. Draft diagram created in Doubao with AI‐assisted layout suggestions (accessed July 2025). Final image refined and annotated by the authors. (b) Environmental factors such as high altitude and intense UV radiation in Sichuan Tibetan regions induced bud sport mutations in TBY, significantly altering carotenoid, sugar, and lignin contents in the fruit flesh.

## CONCLUSION

Bud sports generate abundant genetic variations in fruit trees and have become one of the most important breeding approaches for fruit trees. Nevertheless, research on the molecular mechanisms of bud sport remains limited. To explore the molecular mechanism by which bud sports modify fruit quality in loquat, this study systematically characterized genetic variation features between the bud‐sport mutant TBW and its wild‐type using a *de novo*‐assembled high‐quality genome. Integrative analyses of comprehensive phenotypic data and multi‐omics data elucidated key metabolites and the underlying molecular mechanisms governing carotenoids, sugar, and phenylpropane metabolic pathways in the bud‐sport loquat. Meanwhile, combined with comparative genomics and gene functional validation, the results confirmed that the homozygous genotype *EjPSY2A* ^*d*^ */EjPSY2A* ^*d*^, converted from the heterozygous *EjPSY2A/EjPSY2A* ^*d*^ locus, constitutes the primary causal factor for the white‐flesh phenotype in TBW. This study provides valuable insights and novel findings for dissecting the heritable molecular mechanisms underlying bud sports in fruit trees, and facilitates a deeper understanding and practical application of bud‐sport‐based breeding.

## MATERIALS AND METHODS

### Plant material and growth condition

The yellow‐fleshed ‘Dongting’ loquat (TBY) and its white‐fleshed mutant (TBW) were discovered in Aba Tibetan and Qiang Autonomous Prefecture of Sichuan Province in 2003. TBY refers to ‘Dongting’ loquat with yellow flesh, an ancient local cultivar widely cultivated in southern China since the Ming Dynasty. The fruit of TBY is oval in shape. Its peel and flesh enter the color transition stage in mid to late April. The fruit development period is approximately 155 days. When fully ripe, both the peel and flesh are orange‐yellow, with medium flesh firmness and a sweet–sour flavor. TBW is a bud sport identified on a major branch of TBY, which is nearly round in shape. Its fruit development period, as well as the color transition stages of peel and flesh, are consistent with those of TBY. When ripe, the peel is yellow, while the flesh is nearly white, soft and delicate, with a sweeter taste. Additionally, our previous results demonstrated that the period after color transition is the critical stage for fruit quality differentiation between TBY and TBW (Li et al., 2017). The two materials were grafted onto loquat rootstocks (*Eriobotrya japonica* L.) in Taixing Town, Xindu District, Chengdu City in 2014. The plants were spaced at 4 m × 5 m intervals. Cultivation and management were both performed following local conventional cultivation practices.

### Determining genome size by flow cytometry

Sufficient quantities of young leaves from TBY and TBW were collected for flow cytometry to evaluate the size of the genome. A total of 0.2 g young leaves from TBY or TBW were cut and placed in a Petri dish, and 1 mL nuclear extraction solution was added to the dish, consisting of 15 mmol·L^−1^ Tris–HCl (pH = 7.5), 20 mmol·L^−1^ KCl, 80 mmol·L^−1^ NaCl, 20 mmol·L^−1^ EDTA‐Na_2_, 2% PVP, and 0.2% TritonX‐100. The leaves were then finely chopped using a double‐sided blade. After incubation for 5 min, the nuclei suspension was obtained by filtering with 300 mesh nylon mesh. The nuclear suspensions of ‘Nipponbare’ (*Oryza Sativa*) were prepared in the same manner. Subsequently, 300 μL of the loquat's nuclei suspension was mixed with an equal volume of the nuclei suspension from the internal reference (Nipponbare). Each mixed suspension (0.5 mL) was combined with 0.5 mL of propidium iodide staining solution (45 mg·L^−1^ RNase A and 10 mg·L^−1^ PI). The mixtures were added to a 1.5 mL centrifuge tube, fully shaken in ice bath, and then dark staining for about 0.5 h. Flow cytometry (Beckman Coulter, Brea, CA, USA) was used to detect the Propidium Iodide (PI) fluorescence excitation intensity of the samples. Three parallel tests were conducted for each sample, collecting over 2000 cells from each group. CytExpert v2.0 (Beckman Coulter, Brea, CA, USA) was used to analyze the data, and the parallel test data with the largest number of cells collected as well as similar morphology of the fluorescence peaks between the sample and internal standard were plotted.

### Genome sequencing and assembly

Total genomic DNA of TBY and TBW was extracted from young fresh leaves. According to the protocols from Illumina (Illumina, San Diego, CA, USA), genomic DNA was then used to generate ~350 bp DNA libraries for genome survey. The library underwent paired‐end sequencing using the Illumina NOVASEQ 6000 platform. The sequencing data were then used to estimate the genome size, heterozygous ratio, and repeat ratio. The loquat genomic DNA was fragmented to prepare a 15–18 kb library using a fully automated nucleic acid shearing instrument (Diagenode, Liege, BEL). The library was then constructed using the SMRTbell Express Template Prep kit 2.0 (Pacific Biosciences, Menlo Park, CA, USA) and sequenced using the PacBio Sequel II platform. Low‐quality reads and sequence adapters were filtered out, and then the genome was assembled by Hifiasm (https://github.com/chhylp123/hifiasm). To assist the genome assembly, we constructed 300–700 bp Hi‐C fragment libraries and sequenced them using the Illumina NOVASEQ 6000 platform. The raw data obtained by Hi‐C sequencing was filtered using HiC‐Pro v2.10.0 (Servant et al., 2015). The clean data was aligned with the initially assembled genome to obtain mapped data. To further improve the genome assembly, we utilized LACHESIS (Burton et al., 2013) to divide, sort and orient the genome sequence followed by manual mapping and inspection, and finally obtain the chromosome‐level genome. The consensus quality values for the two assembled genomes were calculated using Merqury (https://github.com/marbl/merqury). The assembled genomes were subjected to analysis with quarTeT (http://atcgn.com:8080/quarTeT/home.html) for the characterization and visualization.

### Analyses of structural variation and loss of heterozygosity

Structural variations (SVs) between the TBY and TBW genomes were identified by aligning the two genome assemblies using minimap2 (Li, 2018), followed by SV detection using SyRI (Goel et al., 2019). The identified SVs were visualized using plotsr (https://github.com/schneebergerlab/plotsr). Loss of heterozygosity (LOH) regions were identified using BCFTools (https://github.com/samtools/bcftools). The genome was scanned using a 100‐kb sliding window with a 10‐kb step size, and the number of heterozygous SNPs within each window was calculated. Windows containing ≤2 heterozygous SNPs were defined as candidate LOH regions. Adjacent candidate regions separated by ≤10 kb were merged into continuous LOH regions. The total number and length of the resulting LOH regions were calculated. The identified LOH regions were then intersected with the genome annotation using BEDTools (Quinlan & Hall, 2010) to identify genes located within these regions. The distribution of LOH regions and associated genomic features was visualized using ggplot2 (https://ggplot2.tidyverse.org/).

### Repeat sequence and gene annotation

Repeat Modeler2 (Flynn et al., 2020), RECON (Bao & Eddy, 2002), and Repeat Scout (Price et al., 2005) were used for repeat prediction. The predictions were classified using repbase (Jurka et al., 2005), REXdb (Neumann et al., 2019), and Dfam (Wheeler et al., 2012). For transcriptome‐assisted annotation of genes, we collected stems, young leaves, inflorescence, and fruit peel. Subsequently, the RNA extracted from different tissues and organs was mixed in equal amounts and used to determine the full‐length transcriptome by the protocol provided by Oxford Nanopore Technologies. Augustus (Stanke et al., 2008), SNAP (Korf, 2004), and GeMoMa (Keilwagen et al., 2016) were the prediction tools. Transcriptome prediction and gene annotation involved Hisat (Kim et al., 2015), Stringtie (Pertea et al., 2015), and GeneMarkS‐T (Tang et al., 2015). Genome integrity evaluation included BWA alignment (Li & Durbin, 2010), Core Eukaryotic Genes Mapping Approach (CEGMA) analysis (Parra et al., 2007), and BUSCO alignment (Simão et al., 2015). Among them, the BUSCO analysis utilized the embryophyta database, which contains 1614 conserved core genes. The BUSCO v4.0 software was employed to evaluate the completeness of gene prediction (Simão et al., 2015). Non‐coding RNA identification utilized tRNAscan‐SE (Lowe & Eddy, 1997), Rfam (Griffiths‐Jones, 2004), miRbase (Griffiths‐Jones, 2006), and Infernal (Nawrocki & Eddy, 2013). Functional annotation analysis employed Non‐Redundant Protein Database (NR), Evolutionary genealogy of genes: Non‐supervised Orthologous Groups (EggNOG), Gene Ontology (GO), Kyoto Encyclopedia of Genes and Genomes (KEGG), SWISS‐PROT, and Pfam databases.

### Observation of fruit phenotype

According to previous studies, TBY and TBW share identical phenological stages, and the period within 7 days after flesh color transition in TBY represents the critical window for fruit quality divergence. Therefore, we randomly collected twenty fruits from each genotype at 1 week prior to flesh color transition (143 DAP), at the color transition stage (150 DAP), and at the fruit‐ripening stage (155 DAP), which were designated as S1, S2, and S3 stages, respectively. Fruits with uniform appearance, size, and maturity were selected for phenotypic evaluation, with three biological replicates performed. Fruit images were captured under identical conditions, and fruit color parameters were measured according to the method described previously (Song, Liu, et al., 2022). Briefly, L* (brightness), a* (redness/greenness), b* (yellowness/blueness), chroma (C*), and hue angle (h°) values were determined using an NH‐200 colorimeter (Sine Image, Shenzhen, Guangdong, China). Measurements were performed at multiple positions on each fruit surface, and the average value was calculated for each fruit. Fruit firmness was measured using a GY‐3 firmness tester (Tuopuyunnong, Hangzhou, Zhejiang, China) after instrument calibration. After phenotype evaluation, the peel and core were removed, and the flesh was immediately chopped in an ice bath, frozen in liquid nitrogen, and stored at −80°C for subsequent analyses.

### Transcriptome analysis

RNA was extracted from flesh tissues of TBY and TBW fruits collected at different developmental stages after phenotypic observation for transcriptome sequencing. The tissue used in the RNA‐Seq analysis included fleshes from TBY and TBW at 1 week prior to flesh color transition (143 DAP), at the color transition stage (150 DAP), and at the fruit‐ripening stage (155 DAP), which were designated as TBY/TBW‐S1, TBY/TBW‐S2, and TBY/TBW‐S3, respectively (Table S15). Meanwhile, to facilitate comparisons of tissue‐specific expression patterns of different genes, fruit peel, inflorescence, stem and leaf tissues of TBY were also sampled for transcriptome sequencing, which were designated as TBY‐peel, TBY‐inf, TBY‐stem and TBY‐leaf, respectively (Table S15). Three independent biological replicates were prepared for each tissue sample in all transcriptome sequencing libraries. Transcriptome sequencing was performed using the Illumina NOVASEQ 6000 platform. RNA reads were aligned to the loquat reference genome with the accession number of GWHAAZU00000000 (https://ngdc.cncb.ac.cn/gwh/documents), using HISAT2 (Kim et al., 2019) and assembled and quantified using String Tie (Pertea et al., 2015). The transcriptome sequencing data presented in the study are deposited in the National Genomics Data Center (NGDC) repository (https://ngdc.cncb.ac.cn/gsa), accession number CRA014295. DESeq2 (Love et al., 2014) was utilized to identify differentially expressed genes (DEGs) among the loquat flesh samples. DEGs meeting the criteria of |log_2_ Fold Change| ≥1 and FDR <0.01 were considered and annotated in GO and KEGG databases. Enrichment analysis of DEGs was performed using Omicshare (https://www.omicshare.com/). The expression levels were determined by calculating the number of fragments per kilobase of transcript per million fragments mapped (FPKM). Principal component analysis based on gene expression levels was conducted using Omicshare (https://www.omicshare.com/). A local database was established with the TBY and TBW genomes, and key genes involved in the carotenoid, sugar, and phenylpropanoid metabolism pathway in loquat were identified using BlastP following the method of Su et al. (2023).

### Validation of gene expression by RT‐qPCR

To validate the reliability of the RNA‐seq data, 48 highly expressed DEGs associated with carotenoid, sugar, and phenylpropanoid metabolism pathways were selected for RT‐qPCR analysis. Total RNA was extracted from each sample using the RNAprep Pure Polysaccharide and Polyphenol Plant Total RNA Extraction Kit (DP441, TIANGEN, Beijing, China) according to the manufacturer's instructions. The quality and concentration of RNA samples were evaluated before reverse transcription. First‐strand cDNA was synthesized from qualified RNA samples and used as templates for RT‐qPCR analysis. Quantitative PCR was performed using the blastaq™ 2 × qPCR Master Mix Kit (G891, ABM, Vancouver, Canada) on a LightCycler® 480 Real‐Time PCR System (Roche Diagnostics, Basel, Switzerland). Each reaction was performed in a final volume of 20 μL, containing 10 μL of 2 × qPCR Master Mix, 0.4 μL of forward primer (10 μM), 0.4 μL of reverse primer (10 μM), 2 μL of diluted cDNA template, and 7.2 μL of nuclease‐free water. The amplification program was performed as follows: initial denaturation at 95°C for 3 min, followed by 40 cycles of 95°C for 10 s and 60°C for 30 s. A melting curve analysis was conducted after amplification to verify the specificity of the PCR products. The relative expression levels of the selected genes were calculated using the 2^−ΔΔCt method and normalized against the expression level of the reference gene *EjTUB (Eja09G007630)*. Three independent biological replicates were analyzed for each sample, with each replicate containing three technical replicates. The information of the genes selected for RT‐qPCR validation and the corresponding oligonucleotide primer sequences are provided in Table S17.

### Detection and analysis of metabolites

The present study employed multiple analytical methods to investigate the metabolites in loquat flesh. The contents of carotenoids, chlorophyll a, and chlorophyll b were determined using an acetone extraction method following the method of Song, Liu, et al. (2022). The components of carotenoid, sugar, and phenylpropanoid, as well as the total content of abscisic acid, were detected using HPLC‐MS following the method of Shu et al. (2023). Furthermore, differentially accumulated metabolites (DAM) related to sugar, carotenoid, and phenylpropanoid were screened using specific criteria. The screening index for sugar components was *P <* 0.05, fold change ≥1.5 or ≤0.67, while |log2 fold change| ≥1 and *P*‐value <0.05 were used as the screening index for carotenoid and phenylpropanoid components. Key DAMs and DEGs were visualized using TBtools (Chen et al., 2020) and Adobe Illustrator 2022.

### Screening of key genes related to the metabolites with WGCNA

WGCNA was performed using the cloud platform of Metaware Company (https://cloud.metware.cn/), where the Pearson correlation coefficient was calculated for the transcriptome data, components of carotenoid, sugar, phenylpropanoid, and some fruit quality indicators.

### Transient overexpression in loquat flesh

The full‐length coding sequences of *EjPSY2A* and *EjNCED2* were individually cloned into the pBI121‐35S‐3×FLAG plasmid to construct the overexpression vectors. The transient transformation assay was conducted as described previously (Huang et al., 2024). In brief, the recombinant plasmids were introduced into *Agrobacterium tumefaciens* GV3101(psoup‐p19) using the freeze–thaw method. Subsequently, the bacterial suspension was cultured at 28°C with shaking at 180 rpm until the OD value reached between 0.8 and 1.0. The overexpressed recombinant plasmids were injected into the flesh of loquat. Three days later, an injection was administered again at the same injection site. Seven days later, samples were taken from the injection sites of the fruits for measurement. Primers used for vector construction are listed in Table S2.

### Genotyping and sequence alignment of *EjPSY2A*

The *PSY2A* locus of TBY and TBW was genotyped following the method previously described (Song, Liu, et al., 2022). Homologous genes of *PSY2A* in ‘Jiefangzhong’ (Su et al., 2021) and ‘Seventh star’ (Jiang, An, et al., 2020) were obtained through BlastP. Subsequently, DNAMAN version 9.0 (https://www.lynnon.com) was used to compare the DNA sequences in different loquat materials. LINKVIEW2 (https://yangjianshun.github.io/LINKVIEW2) and GSDS 2.0 (https://gsds.gao‐lab.org) were employed to perform the gene structure.

### Lignin staining and content determination

The lignin in the mature flesh of loquat was stained with phloroglucinol‐HCl and observed using a binocular stereomicroscope (Leica, Heerbrugg, Switzerland) as described previously (Zhu et al., 2024). The content of lignin in flesh was determined by a kit (AKSU010U, Boxbio, Beijing).

## Author Contributions

HS, DC and RG conceived the project and its components. HS, KZ and JC wrote the paper. GJ, SS, HX, JL MT collected samples. YP and ML supervised the study. HS, KZ and TL conducted the experimental work and analyzed the data. JC and ML reviewed the original manuscript. All authors read and approved the final manuscript.

## Conflict of Interest Statement

The authors declare no competing interests.

## Supporting information


**Table S1:** Survey results of TBY and TBW.
**Table S2:** Estimated genome size by flow cytometry.
**Table S3:** Sequencing data output.
**Table S4:** Length distribution of reads obtained by circular consensus sequencing.
**Table S5:** Results of Hi‐C assisted genome assembly.
**Table S6:** Statistics of TBY and TBW genome assemblies.
**Table S7:** Assessing the integrity of gene prediction.
**Table S8:** Global statistical comparison between loquat genomes assembled in this study and available loquat genomes.
**Table S9:** Results of gene annotation.
**Table S10:** Results of annotation of gene function.
**Table S11:** Regions of SNPs and InDels detected in TBW.
**Table S12:** Genome‐wide synteny and structural variation between TBY and TBW.
**Table S13:** Genome‐wide summary of LOH blocks across TBW chromosomes.
**Table S14:** Information of the 20 randomly selected SNPs in TBW.
**Table S15:** Paired‐end transcriptome sequencing data for ten tissues of TBY and TBW.
**Table S16:** Number of differentially expressed genes between TBY and TBW during flesh color conversion.
**Table S17:** Primers for fluorescence quantification and expression profiling.

**Data S1:** The top 30 GO terms resulting from the enrichment analysis of DEGs during the S1 stage between TBY and TBW.
**Data S2:** The top 30 GO terms resulting from the enrichment analysis of DEGs during the S2 stage between TBY and TBW.
**Data S3:** The top 30 GO terms resulting from the enrichment analysis of DEGs during the S3 stage between TBY and TBW.
**Data S4:** Content of carotenoid components in flesh of TBY and TBW at different developmental stages.
**Data S5:** Genes related to carotenoid and abscisic acid biosynthesis, and their expression levels in different organs and tissues of loquat.
**Data S6:** Content of sugar components in flesh of TBY and TBW at different developmental stages.
**Data S7:** Genes related to sucrose biosynthesis, and their expression levels in different organs and tissues of loquat.
**Data S8:** Content of phenylpropanoid components in flesh of TBY and TBW at different developmental stages.
**Data S9:** Genes related to phenylpropanoid biosynthesis, and their expression levels in different organs and tissues of loquat.

**Figure S1:** Overview of the experimental design. Schematic representation of the overall experimental strategy employed in this study. Two loquat genotypes, the yellow‐fleshed Dongting loquat (TBY) and its spontaneous white‐fleshed mutant (TBW), were compared across three fruit developmental stages (S1: 143 days after pollination, S2: 150 DAP, S3: 155 DAP). Flesh tissues were sampled and subjected to two major analytical branches: (1) Phenotypic and cellular analysis, including flesh color measurement, fruit firmness determination, and lignin histochemical staining and quantification; (2) Multi‐omics analysis, comprising genomics (de novo genome assembly and annotation, genotyping of the key carotenogenic gene *EjPSY2A*, and promoter heterozygosity analysis of key metabolic genes), transcriptomics (RNA‐seq profiling, differentially expressed gene identification, weighted gene co‐expression network analysis, and RT‐qPCR validation of 51 candidate genes), and metabolomics (HPLC‐MS‐based quantification of carotenoids, sugars, phenylpropanoids, and abscisic acid content). Functional validation was performed via Agrobacterium‐mediated transient overexpression of *EjPSY2A* and *EjNCED2* in loquat flesh.
**Figure S2:** K‐mer and CCS output data length distribution of TBY and TBW. (A, B) K‐mer distribution of TBY and TBW. (C, D) CCS output data length distribution of TBY and TBW. The abscissa represents the length of CCS (bp). The left ordinate represents the number of CCS, corresponding to the green bar chart. The ordinate on the right represents the total number of bases (Mb) contained in CCS larger than the corresponding length, corresponding to the black curve. The red dashed line indicates the N50 length of the CCS.
**Figure S3:** Nuclei of two loquat materials (TBY and TBW) were sorted by flow cytometry using ‘Nipponbare’ (*Oryza Sativa*) as internal standard. The abscissa represents the relative intensity of the fluorescence signal, and the ordinate is the number of cells.
**Figure S4:** Chromosome‐level distribution of unresolved regions and associated genes in TBY and TBW genome assemblies. The locations of scaffold gaps relative to chromosome ends are shown for the TBY and TBW genome assemblies. Orange squares indicate scaffold gaps, while blue triangles indicate telomeres. TBY contains 33 scaffold gaps, whereas TBW contains 30 scaffold gaps.
**Figure S5:** Genome‐wide synteny and structural variation landscape between TBY and TBW genomes. Genome‐wide collinearity and structural variations between the TBY and TBW genomes were identified using SyRI (https://github.com/schneebergerlab/syri). Blue and orange lines represent the TBW and TBY chromosomes, respectively, while gray regions indicate syntenic regions. Colored connections represent structural variations, including inversions, translocations, and duplications.
**Figure S6:** Taking *Eja09G004400* (*EjC4H8*) as an example, sequencing peaks of the amplified products at five SNP sites. The red circle indicates the SNP site detected in TBW. Lines of different colors represent different bases. A single peak indicates that the site is homozygous, while the double peak indicates that the site is heterozygous.
**Figure S7:** KEGG pathway enrichment analysis of SNPs (A) and InDels (B) between TBY and TBW. The color of each bubble corresponds to the *P*‐value, while bubble size reflects the number of genes enriched in that pathway.
**Figure S8:** Heatmap of correlation among main color difference indexes, pigment components and abscisic acid content in TBY flesh. One and two asterisks indicate statistical significance by Student's *t*‐tests at 0.05 and 0.01 levels, respectively.
**Figure S9:** Based on the results of comparative genomic analysis, it was found that there was a 321 bp deletion in the *PSY2A* gene of TBW. *Eja11G011710.1*, *Ej00015134*, *EjaChr11G011600.1* and *EVM0037308.1* are *PSY2A* genes of TBY, ‘Jiefangzhong’, TBW and ‘Seventh Star’, respectively.
**Figure S10:** The results of qRT‐PCR verification of 16 highly expressed DEGs involved in carotenoid and abscisic acid metabolism pathway. PSY: phytoenesynthase; PDS: ζ‐carotene desaturase; ZDS: Zeta‐carotene desaturase; CRTISO: carotenoid isomerase; LYCB: β‐ionone‐forming lycopene cyclase; CYP97A: cytochrome P450‐type monooxygenase 97A; CYP97C: cytochrome P450‐type monooxygenase 97C; ZEP: zeaxanthin epoxidase; NCED: 9‐cis‐epoxycarotenoid dioxygenase; ABA: NAD(P)‐binding Rossmann‐fold superfamily protein; AAO: ascorbic acid oxidase; CYP707A: Abscisic acid 8′‐hydroxylase. One or two asterisks indicate statistical significance by Student's t‐tests at 0.05 and 0.01 levels, respectively.
**Figure S11:** Correlation analysis of RNA‐Seq data and RT‐qPCR expression levels of 16 highly expressed DEGs involved in carotenoid and abscisic acid metabolism pathways. The correlation between RNA‐seq and RT‐qPCR log_2_(fold change) values was evaluated using Pearson's correlation coefficient (for linear relationships) and Spearman's rank correlation coefficient (for monotonic relationships), with the significance level set at α = 0.05.
**Figure S12:** The results of qRT‐PCR verification of 20 highly expressed DEGs involved in sugar metabolism pathway. One or two asterisks indicate statistical significance by Student's *t*‐tests at 0.05 or 0.01 levels, respectively. SUS: sucrose synthase; NINV: alkaline/neutral invertase; SDH: sorbitol dehydrogenase; FRK: fructokinase; HxK: hexokinase; SPS: sucrose phosphate synthase; GPI: glucose‐6‐phosphate isomerase; PGM: phosphoglucomutase. One or two asterisks indicate statistical significance by Student's t‐tests at 0.05 and 0.01 levels, respectively.
**Figure S13:** Correlation analysis of RNA‐Seq data and RT‐qPCR expression levels of 20 highly expressed DEGs involved in sugar metabolism pathway. The correlation between RNA‐seq and RT‐qPCR log_2_(fold change) values was evaluated using Pearson's correlation coefficient (for linear relationships) and Spearman's rank correlation coefficient (for monotonic relationships), with the significance level set at α = 0.05.
**Figure S14:** The results of qRT‐PCR verification of 12 highly expressed DEGs involved in phenylpropanoid metabolism pathway. PAL: phenylalanine ammonia‐lyase; C4H: cinnamate‐4‐hydroxylase; 4CL: 4‐coumarate‐CoA ligase; LAC: laccase; CHI: chalcone isomerase; F3H: flavonoid 3‐hydroxylase; DFR: dihydroflavonol 4‐reductase; LDOX: leucoanthocyanidin dioxygenase; ANR: anthocyanidin reductase; LAR: leucoanthocyanidin reductase. One or two asterisks indicate statistical significance by Student's *t*‐tests at 0.05 or 0.01 levels, respectively.
**Figure S15:** Correlation analysis of RNA‐Seq data and RT‐qPCR expression levels of 12 highly expressed DEGs involved in sugar metabolism pathway. The correlation between RNA‐seq and RT‐qPCR log_2_(fold change) values was evaluated using Pearson's correlation coefficient (for linear relationships) and Spearman's rank correlation coefficient (for monotonic relationships), with the significance level set at α = 0.05.
**Figure S16:** Genomic landscape of LOH regions flanking the *EjPSY2A* locus on Chr11 in TBW. The *EjPSY2A* gene (black bar, ~10.8 Mb) is located within a heterozygous segment (gray) on Chr11, flanked by two distinct LOH blocks (red): an upstream LOH region spanning 10.51–10.72 Mb and a downstream LOH region spanning 10.86–10.98 Mb. Chr11 position is shown in base pairs (bp).

## Data Availability

The chromosome‐level genome assemblies of two loquat materials were submitted to NCBI (https://www.ncbi.nlm.nih.gov/) under the accession numbers JBIENN000000000 and JBIENO000000000, respectively. The genome assembly files (FASTA) and the annotation files (GFF) were also deposited in China National Center For Bioinformation (https://ngdc.cncb.ac.cn/gwh/) with the accession numbers GWHETRZ00000000.1 and GWHETSA00000000.1, respectively. The RNA‐seq data presented in the study have been deposited in the National Geophysical Data Center (NGDC, https://ngdc.cncb.ac.cn/gsa) with the accession number of CRA011296.
