# Supplementary material for: Two nearly complete genomes reveal the molecular mechanism underlying the significant fruit quality differences caused by bud sport between yellow and white‐fleshed loquat (Eriobotrya japonica L.)
Source: Plant J. 2026 Nov 4;128(3):e71212. doi: 10.1111/tpj.71212 (PMC13736142; doi:10.1111/tpj.71212)
Supplement: Supplementary file 1 [file TPJ-128-0-s003.docx]

**Fig. S1** Overview of the experimental design. Schematic representation of the overall experimental strategy employed in this study. Two loquat genotypes, the yellow-fleshed Dongting loquat (TBY) and its spontaneous white-fleshed mutant (TBW), were compared across three fruit developmental stages (S1: 143 days after pollination, S2: 150 DAP, S3: 155 DAP). Flesh tissues were sampled and subjected to two major analytical branches: (1) Phenotypic and cellular analysis, including flesh color measurement, fruit firmness determination, and lignin histochemical staining and quantification; (2) Multi-omics analysis, comprising genomics (de novo genome assembly and annotation, genotyping of the key carotenogenic gene *EjPSY2A*, and promoter heterozygosity analysis of key metabolic genes), transcriptomics (RNA-seq profiling, differentially expressed gene identification, weighted gene co-expression network analysis, and RT-qPCR validation of 51 candidate genes), and metabolomics (HPLC-MS-based quantification of carotenoids, sugars, phenylpropanoids, and abscisic acid content). Functional validation was performed via Agrobacterium-mediated transient overexpression of *EjPSY2A* and *EjNCED2* in loquat flesh.

**Fig. S2** K-mer and CCS output data length distribution of TBY and TBW. **(A and B)** K-mer distribution of TBY and TBW. **(C and D)** CCS output data length distribution of TBY and TBW.The abscissa represents the length of CCS (bp). The left ordinate represents the number of CCS, corresponding to the green bar chart. The ordinate on the right represents the total number of bases (Mb) contained in CCS larger than the corresponding length, corresponding to the black curve. The red dashed line indicates the N50 length of the CCS.

**Fig. S3** Nuclei of two loquat materials (TBY and TBW) were sorted by flow cytometry using ‘Nipponbare’ (*Oryza Sativa*) as internal standard. The abscissa represents the relative intensity of the fluorescence signal, and the ordinate is the number of cells.

**Fig. S4** Chromosome-level distribution of unresolved regions and associated genes in TBY and TBW genome assemblies.The locations of scaffold gaps relative to chromosome ends are shown for the TBY and TBW genome assemblies. Orange squares indicate scaffold gaps, while blue triangles indicate telomeres. TBY contains 33 scaffold gaps, whereas TBW contains 30 scaffold gaps.

**Fig. S5** Genome-wide synteny and structural variation landscape between TBY and TBW genomes. Genome-wide collinearity and structural variations between the TBY and TBW genomes were identified using SyRI ([https://github.com/schneebergerlab/syri](https://link.gitcode.com/?target=https://github.com/schneebergerlab/syri&from=https://gitcode.com/gh_mirrors/sy/syri/?utm_source=gitcode_aigc_v1_t1&index=bottom&type=card&&uuid_tt_dd=10_19743106030-1787473945096-971274&isLogin=9&from_id=155628610&from_link=3749f8a6249e41bb54c071fb5fd929f2&lang=zh&theme=white)). Blue and orange lines represent the TBW and TBY chromosomes, respectively, while gray regions indicate syntenic regions. Colored connections represent structural variations, including inversions, translocations, and duplications.

**Fig. S6** Taking *Eja09G004400* (*EjC4H8*) as an example, sequencing peaks of the amplified products at five SNP sites. The red circle indicates the SNP site detected in TBW. Lines of different colors represent different bases. A single peak indicates that the site is homozygous, while the double peak indicates that the site is heterozygous.

**Fig. S7** KEGG pathway enrichment analysis of SNPs (A) and InDels (B) between TBY and TBW. The color of each bubble corresponds to the p‑value, while bubble size reflects the number of genes enriched in that pathway.

**Fig. S8** Heatmap of correlation among main color difference indexes, pigment components and abscisic acid content in TBY flesh. One and two asterisks indicate statistical significance by Student’s ***t***-tests at 0.05 and 0.01 levels, respectively.

**Fig. S9** Based on the results of comparative genomic analysis, it was found that there was a 321 bp deletion in the *PSY2A* gene of TBW. *Eja11G011710.1*, *Ej00015134*, *EjaChr11G011600.1* and *EVM0037308.1* are *PSY2A* genes of TBY, ‘Jiefangzhong’, TBW and ‘Seventh Star’, respectively.

**Fig. S10** The results of qRT-PCR verification of 16 highly expressed DEGs involved in carotenoid and abscisic acid metabolism pathway. PSY: phytoenesynthase; PDS: ζ-carotene desaturase; ZDS: Zeta-carotene desaturase; CRTISO: carotenoid isomerase; LYCB: β-ionone-forming lycopene cyclase; CYP97A: cytochrome P450-type monooxygenase 97A; CYP97C: cytochrome P450-type monooxygenase 97C; ZEP: zeaxanthin epoxidase; NCED: 9-cis-epoxycarotenoid dioxygenase; ABA: NAD(P)-binding Rossmann-fold superfamily protein; AAO: ascorbic acid oxidase; CYP707A: Abscisic acid 8'-hydroxylase. One or two asterisks indicate statistical significance by Student’s t-tests at 0.05 and 0.01 levels, respectively.

**Fig. S11** Correlation analysis of RNA-Seq data and RT-qPCR expression levels of 16 highly expressed DEGs involved in carotenoid and abscisic acid metabolism pathways. The correlation between RNA-seq and RT-qPCR log_2_(fold change) values was evaluated using Pearson's correlation coefficient (for linear relationships) and Spearman's rank correlation coefficient (for monotonic relationships), with the significance level set at α = 0.05.

**Fig. S12** The results of qRT-PCR verification of 20 highly expressed DEGs involved in sugar metabolism pathway. One or two asterisks indicate statistical significance by Student’s ***t***-tests at 0.05 or 0.01 levels, respectively. SUS: sucrose synthase; NINV: alkaline/neutral invertase; SDH: sorbitol dehydrogenase; FRK: fructokinase; HxK: hexokinase; SPS: sucrose-phosphate synthase; GPI: glucose-6-phosphate isomerase; PGM: phosphoglucomutase. One or two asterisks indicate statistical significance by Student’s t-tests at 0.05 and 0.01 levels, respectively.

**Fig. S13** Correlation analysis of RNA-Seq data and RT-qPCR expression levels of 20 highly expressed DEGs involved in sugar metabolism pathway. The correlation between RNA-seq and RT-qPCR log_2_(fold change) values was evaluated using Pearson's correlation coefficient (for linear relationships) and Spearman's rank correlation coefficient (for monotonic relationships), with the significance level set at α = 0.05.

.

**Fig. S14** The results of qRT-PCR verification of 12 highly expressed DEGs involved in phenylpropanoid metabolism pathway. PAL: phenylalanine ammonia-lyase; C4H: cinnamate-4-hydroxylase; 4CL: 4-coumarate-CoA ligase; LAC: laccase; CHI: chalcone isomerase; F3H: flavonoid 3-hydroxylase; DFR: dihydroflavonol 4-reductase; LDOX: leucoanthocyanidin dioxygenase; ANR: anthocyanidin reductase; LAR: leucoanthocyanidin reductase. One or two asterisks indicate statistical significance by Student’s ***t***-tests at 0.05 or 0.01 levels, respectively.

**Fig. S15** Correlation analysis of RNA-Seq data and RT-qPCR expression levels of 12 highly expressed DEGs involved in sugar metabolism pathway. The correlation between RNA-seq and RT-qPCR log_2_(fold change) values was evaluated using Pearson's correlation coefficient (for linear relationships) and Spearman's rank correlation coefficient (for monotonic relationships), with the significance level set at α = 0.05.

**Figure S16** Genomic landscape of LOH regions flanking the *EjPSY2A* locus on Chr11 in TBW.

The *EjPSY2A* gene (black bar, ~10.8 Mb) is located within a heterozygous segment (grey) on Chr11, flanked by two distinct LOH blocks (red): an upstream LOH region spanning 10.51–10.72 Mb and a downstream LOH region spanning 10.86–10.98 Mb. Chr11 position is shown in base pairs (bp).
